# Nanosensor Detection of an Immunoregulatory Tryptophan Influx/Kynurenine Efflux Cycle

**DOI:** 10.1371/journal.pbio.0050257

**Published:** 2007-09-25

**Authors:** Thijs Kaper, Loren L Looger, Hitomi Takanaga, Michael Platten, Lawrence Steinman, Wolf B Frommer

**Affiliations:** 1 Department of Plant Biology, Carnegie Institution, Stanford, California, United States of America; 2 Department of Neuro-oncology, University Hospital of Heidelberg, INF 400, Heidelberg, Germany; 3 German Cancer Research Center, INF 280, Heidelberg, Germany; 4 Department of Neurology and Neurological Sciences, Stanford University, Stanford, California, United States of America; Stanford University, United States of America

## Abstract

Mammalian cells rely on cellular uptake of the essential amino acid tryptophan. Tryptophan sequestration by up-regulation of the key enzyme for tryptophan degradation, indoleamine 2,3-dioxygenase (IDO), e.g., in cancer and inflammation, is thought to suppress the immune response via T cell starvation. Additionally, the excreted tryptophan catabolites (kynurenines) induce apoptosis of lymphocytes. Whereas tryptophan transport systems have been identified, the molecular nature of kynurenine export remains unknown. To measure cytosolic tryptophan steady-state levels and flux in real time, we developed genetically encoded fluorescence resonance energy transfer nanosensors (FLIPW). The transport properties detected by FLIPW in KB cells, a human oral cancer cell line, and COS-7 cells implicate LAT1, a transporter that is present in proliferative tissues like cancer, in tryptophan uptake. Importantly, we found that this transport system mediates tryptophan/kynurenine exchange. The tryptophan influx/kynurenine efflux cycle couples tryptophan starvation to elevation of kynurenine serum levels, providing a two-pronged induction of apoptosis in neighboring cells. The strict coupling protects cells that overproduce IDO from kynurenine accumulation. Consequently, this mechanism may contribute to immunosuppression involved in autoimmunity and tumor immune escape.

## Author Summary

Although regulated suppression of the immune system prevents autoimmunity and is important during pregnancy to protect the fetus or after organ transplant to block graft rejection, it can be harmful if co-opted by tumors to escape detection. T cells of the immune system normally recognize and destroy abnormal cells, including cancerous and grafted tissues. This process requires the amino acid tryptophan. Foreign grafts and cancer cells can dampen the immune response by starving T cells of tryptophan through a mechanism involving uptake and conversion to kynurenines using an enzyme called indoleamine-2,3-dioxygenase (IDO). Independent of tryptophan starvation, kynurenines induce T cell death when they are excreted through an unknown mechanism. We constructed fluorescent nanosensors that report tryptophan levels in two immortalized cell lines, COS-7 cells and human oral cancer KB cells. Using single-cell, real-time imaging analysis, we demonstrate that an L-amino acid transporter protein (LAT1) exchanges tryptophan for its kynurenine degradation products. LAT1 together with IDO forms a metabolic minicycle that couples tryptophan starvation with kynurenine-induced cell death, providing a two-pronged inactivation of neighboring cells. The strict coupling protects the cells that overproduce IDO from accumulating kynurenine. This cycle may contribute to suppression of the immune response in autoimmune diseases and cancer.

## Abstract 3

Genetically encoded FRET nanosensors were developed to measure cytosolic tryptophan steady-state levels and flux in real time.

## Introduction

l-tryptophan is an essential amino acid necessary for protein synthesis in mammalian cells. Moreover, tryptophan is the precursor for the neurotransmitter serotonin, for the hormone melatonin, and contributes to the synthesis of the coenzymes NADH and NADPH. Degradation products of tryptophan have immunoregulatory functions. Mammalian cells cannot synthesize l-tryptophan, and thus depend on transport machineries for its uptake and protein turnover for its production. Because cell growth is strictly dependent on tryptophan serum levels, different cell types compete for this amino acid, for instance in the regulation of the immune response. Identified transporter proteins potentially involved in the uptake of tryptophan in human cells include the Na^+^-independent systems b^0^AT1 [1]; b^0,+^AT [2]; TAT1 [3]; y^+^LAT1 and y^+^LAT2 [4,5]; and LAT1, LAT2, LAT3, and LAT4 [6–9]. Of these, b^0,+^AT, LAT1, LAT2, y^+^LAT1, and y^+^LAT2 are amino acid exchangers; they countertransport cytosolic amino acids for extracellular ones. LAT1 is present in proliferative tissues and, as such, is up-regulated in many human primary tumor cells [10].

Tryptophan can be degraded through the kynurenine pathway, e.g., for the biosynthesis of vitamin B3 or niacin, which is needed for the synthesis of coenzyme NADH or NADPH. The rate-limiting step in this pathway is the opening of the indole ring by indoleamine 2,3-dioxygenase (IDO). Since the discovery that inhibition of IDO induced fetal allograft rejection in mice, the immunosuppressive function of tryptophan catabolism has been well established [11]. One proposed mechanism for the observed immunosuppression is the local depletion of tryptophan, which inhibits adaptive T cell responses by forcing them into growth arrest and inducing apoptosis [12]. As such, the immune escape of many cancer cell types correlates with up-regulated IDO expression and can, in some cases, be overcome by IDO inhibition [13,14]. In addition, products of the kynurenine pathway are immunosuppressive and may provide leads for the treatment of autoimmune disorders such as multiple sclerosis [15]. Kynurenines are natural amino acids found in mammals; however, the transport machinery for their export across the cell membrane is not known.

Traditionally, cellular uptake of molecules has been determined using radiolabeled substrates, and cellular levels have been measured in extracts via liquid chromatography or gas chromatography/mass spectrometry. Both methods are neither time resolved nor specific, and they lack high temporal or cellular/subcellular resolution. Tryptophan is aromatic, binding nonspecifically to many molecules. Given the importance of l-tryptophan for human health, an analytical tool for noninvasive, time-resolved determination of intracellular l-tryptophan levels was deemed desirable.

Fluorescent indicator proteins (FLIPs) are new tools for real-time monitoring of metabolite levels and have been used successfully for monitoring several small molecules in subcompartments in living cells. Typically, the nanosensors consist of a ligand-sensing domain, allosterically coupled to a pair of green fluorescent protein variants capable of fluorescence resonance energy transfer (FRET). FRET requires donor and acceptor fluorophores with overlapping emission and excitation spectra, respectively. After excitation of the donor, energy is transmitted to the acceptor in a nonradiative manner and emitted by the acceptor. The efficiency of this process depends on the distance between and relative orientation of the dipoles of the fluorophores. Ligand binding–induced conformational changes in the sensors result in altered FRET efficiencies, which correlate with the levels of the respective metabolites. Periplasmic binding proteins (PBPs) have been successfully exploited for the construction of FLIPs for imaging of key metabolites such as glucose [16], maltose [17], ribose [18], and glutamate [19]. However, no tryptophan-binding PBPs have been described to date; thus an alternative ligand-sensing scaffold was explored for construction of a tryptophan nanosensor.

In γ-proteobacteria like *Escherichia coli,* transcription of the tryptophan biosynthetic operon is regulated by attenuation [20] and by the inhibitory binding of the tryptophan-activated repressor protein, TrpR, to the *trp* operator [21]. Binding of l-tryptophan to the repressor results in conformational changes that enhance the repressor's affinity for the operator sequence [22]. We have exploited the ligand-induced conformational changes of TrpR for the construction of novel genetically encoded sensors for monitoring of in vivo l-tryptophan levels. First, we demonstrate the applicability of the metabolite FRET nanosensor technology to novel ligand-sensing domains and instigate new methods for the construction of nanosensors for metabolites that are only present inside the cell (and therefore are unlikely to be recognized by evolved PBPs). Second, we use a novel strategy for the optimization of the FRET signal based on the particular topology and conformation of TrpR. Third, we express the optimized FRET tryptophan sensor in KB and in COS-7 cells and find that these cells take up tryptophan by a LAT amino acid exchanger. Finally, we demonstrate that this transporter mediates tryptophan/kynurenine exchange, which, in cooperation with IDO, provides a new metabolic cycle that may contribute to the immune escape of various tumor cells as well as a favorable role of kynurenines in reducing autoimmunity, while at the same time protecting the cells that overproduce IDO from kynurenine accumulation.

## Results

### A Ligand-Binding Scaffold for l-Tryptophan

**Figure 1:**
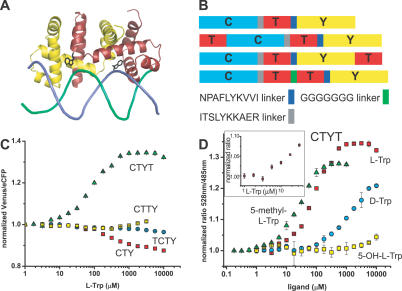
Tryptophan Sensors Based on the E. coli Tryptophan Repressor TrpR (A) TrpR dimer (yellow, red) in complex with l-tryptophan (black) bound to the *trp* operator (green, blue) ([28]). (B) FLIPW variants. (C) Normalized FRET ratio change of FLIPW-CTY (red squares), FLIPW-TCTY (cyan circles), FLIPW-CTYT (green triangles), and FLIPW-CTTY (yellow squares) in the presence of l-tryptophan. (D) Normalized FRET ratio change of FLIPW-CTYT in presence of l-tryptophan (red squares), d-tryptophan (cyan circles), 5-hydroxy-l-tryptophan (yellow squares), and 5-methyl-l-tryptophan (green triangles). Inset in (D) highlights the response of FLIPW-CTYT to tryptophan in the low micromolar range. Ratio is defined as fluorescence intensity quotient of emission at 528 nm/485 nm.

The E. coli tryptophan operon repressor TrpR is an all-helical polypeptide of 108 amino acids organized into 6 α helices. This polypeptide forms a dimer that selectively binds two molecules of l-tryptophan with micromolar affinity (Figure 1A) [23]. In the active, dimeric conformation of TrpR, five of the six helices in each polypeptide are involved in intermolecular contacts [24]. With both chains contributing to each tryptophan-binding site, two TrpR polypeptides are necessary to form the two functional intermolecular binding sites (Figure 1A). In the absence of tryptophan, a part of TrpR is unfolded [25], which likely corresponds to the helix-turn-helix motifs that form the DNA-reading heads. Crystallographic analysis shows that the helix-turn-helix motif undergoes structural rearrangements upon binding of tryptophan [22], and the motif's flexibility is essential for the recognition of operator sequences [26]. In addition, tryptophan binding results in a shift of the relative distance and orientation of the N and C termini of each repressor polypeptide with respect to one another [22], which we detected as a change in FRET when TrpR was fused to a FRET fluorophore pair. The E. coli tryptophan repressor gene was sandwiched between enhanced cyan fluorescent protein (eCFP) and Venus (a yellow fluorescent protein variant) coding sequences (Figure 1B). Production of the translated fusion product FLIPW-CTY (CTY: eCFP-TrpR-VenusYFP) in E. coli was readily detected by recording the emission spectrum of the eCFP-Venus FRET signal in cell cultures. When eCFP was excited, significant energy transfer to Venus was detected, resulting in a Venus/eCFP ratio of 4 (Table S1). Addition of l-tryptophan decreased FRET efficiency of the purified protein, visible as an increase in eCFP emission intensity and a concomitant decrease in Venus fluorescence intensity, resulting in a 10% reduction in the Venus/eCFP ratio (Figure 1C). FLIPW-CTY bound l-tryptophan with an apparent dissociation constant (*K*_d_) of 220 ± 20 μM, which is about one order of magnitude higher compared with unmodified TrpR as measured by equilibrium dialysis [23]. Small molecules are known to quench fluorophore emission efficiently due to nonspecific interactions [27]. To exclude that the negative ratio change observed for FLIP-CTY is due to unspecific effects, the FRET response was measured in the presence of d-tryptophan. Compared with l-tryptophan, unmodified TrpR has a 20-fold reduced affinity for d-tryptophan [23]. Titration of FLIPW-CTY with d-tryptophan resulted in a decrease of the FRET ratio at about 5-fold higher concentrations than l-tryptophan (Figure S1). Because d- and l-tryptophan would be expected to have the same quenching properties, this strongly suggests that the decrease in FRET ratio of FLIPW-CTY is due to a specific interaction of the sensor with tryptophan.

### Twin-Cassette FLIPW Nanosensor Variants

The active conformation of TrpR is a dimer, and two tryptophan-binding sites are formed at the dimer interface [28]. Therefore, one assumes that the functional FLIPW-CTY sensor functions as a dimer of two CTY polypeptides, with the four fluorophores being packed tightly together, potentially affecting the binding affinity due to steric hindrance or resulting in signal loss due to averaging of the fluorophore signals. Thus fusing two TrpR molecules to one fluorophore set may give rise to sensors with a single eCFP-Venus pair per sensor, which may have improved sensing characteristics. Three sensor permutations containing two TrpR copies (a and b) in a single gene product were constructed (Figure 1B). In the permutants FLIPW-TCTY (linear arrangement of TrpR^a^-eCFP-TrpR^b^-Venus) and FLIPW-CTYT (linear arrangement of eCFP-TrpR^a^-Venus-TrpR^b^) the distance between the N and C termini of the intercalated green fluorescent protein variants (eCFP in TCTY and Venus in CTYT) corresponds well to the distance between the C terminus of the first TrpR polypeptide (a) and the N terminus of the second TrpR (b) in the dimer (∼22 Å, Figure S2). One of the fluorophores in these variants is therefore rotationally constrained by these attachment points, a fact that is expected to lead to an improvement of the signal change due to decreased conformational averaging as shown for other “insertional” FRET sensors [29,30]. The third variant, FLIPW-CTTY, is a linear fusion in the order eCFP-TrpR^a^-TrpR^b^-Venus. For the construction of FLIPW-CTTY, two copies of the repressor gene were connected by a flexible linker consisting of seven glycine residues and inserted between the fluorophores. This linker was designed to loosely connect the two TrpR proteins without changing the dimer conformation, and is based on a model constructed in Modeller8v1 [31]. Whereas the FRET ratio of FLIPW-CTTY and FLIPW-TCTY changed only slightly when titrated with l-tryptophan, FLIPW-CTYT yielded a substantially improved tryptophan sensor (Figure 1C and 1D). The apparent binding constant of FLIPW-TCTY for l-tryptophan was ∼20 μM, comparable to unmodified TrpR [23]. The ratio change observed for FLIPW-CTTY could not be fitted to a single-site-binding isotherm (see Material and Methods).

When FLIPW-CTYT was titrated with l-tryptophan, an increase in FRET ratio from 2.2 to 2.8 was observed, indicating a significant change in chromophore orientation with respect to FLIPW-CTY. The ratio change observed in vitro for FLIPW-CTYT was +35%. FLIPW-CTYT bound l-tryptophan with an apparent affinity of 100 ± 10 μM. Analogous to the wild-type TrpR, FLIPW-CTYT binds ligands in order of decreasing affinity: l-5-methyl-tryptophan > l-tryptophan > d-tryptophan > l-5-hydroxy-tryptophan (Table S2) [23]. The positive ratio change permits efficient discrimination of quenching effects; thus, FLIPW-CTYT appears better suited for in vivo measurements compared to FLIPW-CTY, which shows a negative ratio change (Table S1). Therefore FLIPW-CTYT was chosen to monitor physiological tryptophan levels in mammalian cells (dynamic range ∼ 15 μM to 1 mM).

### Molecular Modeling of FLIPW Sensors

**Figure 2:**
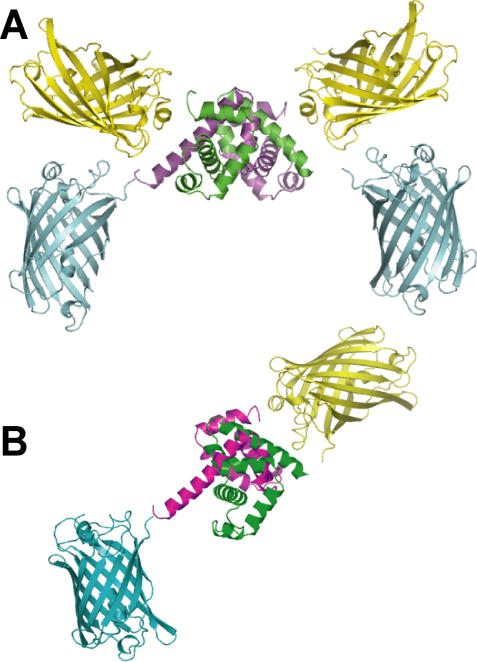
Structural Models of FLIPW-CTY and FLIPW-CTYT Tryptophan Sensors TrpR: green, magenta, eCFP: blue, and Venus: yellow. (A) Dimer of two FLIPW-CTY chains resulting in a TrpR dimer that can bind tryptophan. (B) FLIPW-CTYT monomer. The relative positions of the fluorophore proteins with respect to the TrpR dimer are speculative.

Molecular modeling was performed to rationalize the observed FRET signal changes. The original sensor, FLIPW-CTY, is predicted to dimerize, resulting in an antiparallel arrangement of the TrpR polypeptides, thus resulting in two sets of eCFP and Venus fluorophores in close vicinity at both sides of the TrpR dimer (Figure 2A). In agreement with the close vicinity of the fluorophores, FLIPW-CTY showed the highest FRET efficiency (Table S1). The FLIPW-CTYT sensor is modeled to form a functional TrpR dimer intramolecularly, resulting in a single eCFP and a single Venus molecule per sensor (Figure 2B). FLIPW-CTYT has lower absolute energy transfer efficiency, which is consistent with the greater distance between the fluorophore dipoles. The relative FRET change is higher compared to FLIPW-CTY, probably because of the rigidification of the attachment of the Venus molecule by its fusion to both TrpR monomers. The FLIPW-CTTY and FLIPW-TCTY sensors do not show sufficient ligand-dependent ratio changes to be useful as sensors. Molecular modeling may explain the different responses of the FLIPW-CTYT and FLIPW-TCTY sensors. In FLIPW-CTYT, a fluorophore is attached to the N terminus of TrpR^a^, leading to a different spatial arrangement and rotational probability space compared to FLIPW-TCTY, in which Venus is attached to the C terminus of TrpR^b^. This geometrical difference is presumably transduced into altered dipole orientations in the FLIPW-CTYT sensor (Figure S3).

### Tryptophan Uptake in COS-7 Cell Cultures in 96-Well Microplates

**Figure 3:**
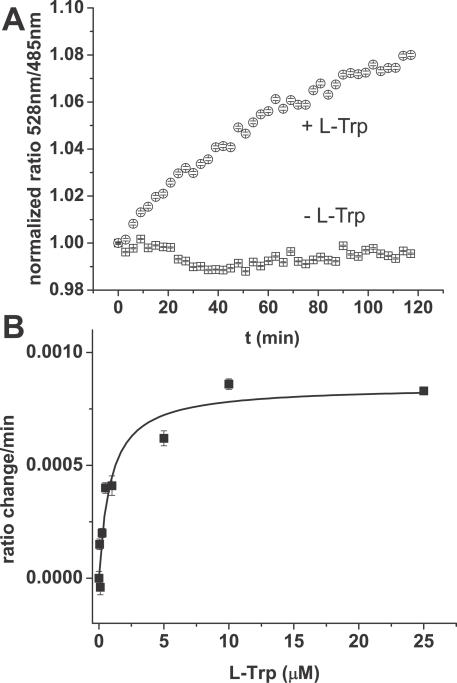
Uptake of Tryptophan by COS-7 Cell Cultures in 96-Well Microplates Monitored with FLIPW-CTYT (A) FRET ratio change of cell cultures in presence of Tyrode's buffer (squares) and 100 μM l-tryptophan in Tyrode's buffer (circles). Data correspond to means ± SE (*n* = 12). (B) Velocity of intracellular FLIPW-CTYT response versus external tryptophan concentration fitted with the Michaelis-Menten equation. Cells were incubated with 0.05, 0.1, 0.25, 0.5, 1, 5, 10, and 25 μM l- tryptophan. Data correspond to means ± SE (*n* = 6). Ratios are defined as fluorescence intensity quotient of emission at 528 nm/485 nm.

To measure tryptophan flux in the cytosol of live cells, COS-7 cell cultures seeded in a 96-well microplate were transiently transfected with pTK222 for cytosolic production of FLIPW-CTYT sensor. Microscopic analysis of transfected cells showed that FLIPW-CTYT was produced in the cytosol. When microwell-grown cells expressing FLIPW-CTYT were incubated in Tyrode's buffer containing tryptophan and analyzed in a microplate reader, an increase in FRET ratio was observed, indicating an increase in cytosolic tryptophan levels as a result of uptake (Figure 3A). The rate in FRET increase depended on the external tryptophan concentration and showed Michaelis-Menten type kinetics with an apparent affinity constant *K*_M_ of 0.88 ± 0.27 μM for combined transport and metabolism (Figure 3B). The FLIPW-CTYT sensor thus appears suitable to study factors influencing tryptophan transport and metabolism and can be used in high-throughput fluorescence-based assays and drug screens.

### Tryptophan Uptake and Exchange in COS-7 Cells Is Mediated by a LAT1-Like Amino Acid Exchanger

**Figure 4:**
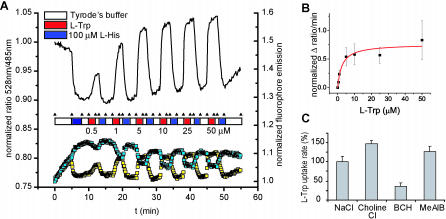
Imaging of Intracellular Tryptophan Levels with FLIPW-CTYT in COS-7 Cells (A) Perfusion of COS-7 cells with various concentrations l-tryptophan (L-Trp) and 100 μM l-histidine (L-His) in Tyrode's buffer. According to the FRET theory, an increase in Venus signal is accompanied by a decrease in eCFP signal. Ratio defined as fluorescence intensity quotient obtained with emission filters 535/40 nm over 480/30 nm. (B) Velocity of intracellular FLIPW-CTYT response versus external tryptophan concentrations used in (A) fitted with the Michaelis-Menten equation. Error bars indicate standard deviation. (C) Effect of Na^+^-ions and inhibitors on the uptake rate of 100 μM l-tryptophan. Error bars indicate standard deviation.

Reported intracellular tryptophan levels in mammalian cells cultivated in batch are in the 0.27–0.60 mM range [32]; thus, they are compatible with the detection range of the FLIPW-CTYT sensor. When COS-7 cells producing cytosolic FLIPW-CTYT sensor were perfused with Tyrode's buffer, the initial FRET ratio (528 nm/485 nm [Venus/eCFP] emission intensity) was stable (Figure 4A). Upon perfusion with 100 μM l-tryptophan, the FRET ratio increased instantaneously, corresponding to rising levels of cytosolic tryptophan. The FRET ratio was stable during subsequent perfusion with buffer, indicating that the cytosolic steady-state tryptophan levels remain constant and that in COS-7 cells tryptophan uniporters like TAT1 [3] do not contribute significantly to transport. When l-histidine was provided in the medium, the eCFP emission increased and Venus emission decreased, evidencing a decrease in FRET efficiency as a result of export of tryptophan from the cytosol. This way, cells could be repeatedly loaded with tryptophan and unloaded using histidine (Figure 4A). By comparison of the starting, minimum, and maximum response levels of the sensor, assuming that the *K*_d_ of the sensor expressed in cells was the same as in vitro, the cytosolic tryptophan concentration was estimated at 340 μM. The affinity of the cells for combined uptake and metabolism of tryptophan was 2.6 ± 1.1 μM (Figure 4B). The system L inhibitor 2-aminobicyclo-(2,2,1)-heptane-2-carboxylic acid (BCH) decreased the uptake of tryptophan, whereas either replacement of sodium with choline or the transport system A inhibitor N-(methylamino)isobutyric acid did not decrease uptake rates (Figure 4C). Na^+^ independence and BCH sensitivity are characteristic of the transport properties of LAT1- and LAT2-like amino acid counterexchangers [6]. Similar to histidine, the larger hydrophobic and aromatic amino acids leucine, isoleucine, valine, methionine, phenylalanine, and tyrosine were able to promote tryptophan export (at 100 μM concentrations, unpublished data), an observation that corresponds best to the reported substrate specificity of LAT1 [33–35]. LAT2 is expressed in the proximal tubule of the kidney [6], so it might be expressed in COS-7 cells. In addition, LAT1 is expressed in various human tumor cell lines [35] and, consequently, is likely to be present in COS-7 cells as well.

### Tryptophan Is Exchanged for Its Kynurenine Degradation Products

**Figure 5:**
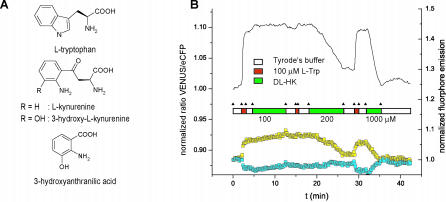
Imaging of Tryptophan-Kynurenine Exchange with FLIPW-CTYT in COS-7 Cells (A) Molecular structures of l-tryptophan, 3-hydroxy-l-kynurenine, and 3-hydroxy-anthranilic acid. (B) Perfusion of COS-7 cells with 100 μM l-tryptophan (L-Trp), 100 μM l-histidine (L-His), and 200–1,000 μM 3-hydroxy-dl-kynurenine (DL-HK) in Tyrode's buffer. According to the FRET theory, an increase in Venus signal is accompanied by a decrease in eCFP signal. Ratio defined as fluorescence intensity quotient obtained with emission filters 535/40 nm over 480/30 nm.

The product of tryptophan conversion by IDO is formylkynurenine (FK), which is, in turn, converted by the enzyme kynurenine formamidase to kynurenine (K) (Figure 5A). The consecutive action of kynurenine-3-hydroxylase produces 3-hydroxy-kynurenine (HK), which is further degraded by kynureninase to hydroxy-anthranilic acid (HAA) (Figure 5A). In vitro, K, HK, and HAA did not result in a FLIPW-CTYT FRET response or interfere with tryptophan binding to the sensor (unpublished data). We tested the effect of extracellular K, HK, and HAA on the intracellular tryptophan levels in tryptophan-preloaded COS-7 cells. Both extracellular K and HK resulted in a reduction of the fluorophore emission ratios in the COS-7 cells, evidencing tryptophan export. The sensor responses for the K- and HK-induced tryptophan were similar (unpublished data), and HK-induced export is shown in Figure 5B. Extracellular HAA did not result in tryptophan export (unpublished data). Thus, the findings are compatible with the role of LAT-like transporters that are present in COS-7 cells as exchangers of tryptophan for its degradation products K and HK. Tryptophan, K, and HK thus appear to be substrates for LAT1 and one may speculate that FK is a substrate as well. Since the substrate specificities of LAT1 and LAT2 differ only in the transport of small amino acids [33,36], we hypothesize that both are capable of tryptophan-kynurenine exchange.

### LAT1 Mediates Tryptophan-Kynurenine Exchange in Human KB Cells

**Figure 6:**
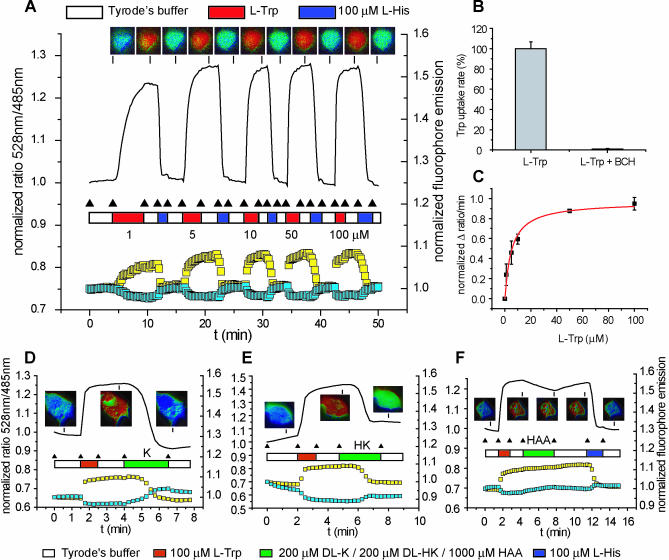
Imaging of Tryptophan Uptake and Exchange with FLIPW-CTYT in Human Oral Carcinoma KB Cells (A) Perfusion of KB cells with various concentrations l-tryptophan (L-Trp) and 100 μM l-histidine (L-His) in Tyrode's buffer. According to the FRET theory, an increase in Venus signal is accompanied by a decrease in eCFP signal. Ratio defined as fluorescence intensity quotient obtained with emission filters 535/40 nm over 480/30 nm. (B) Effect of 5 mM BCH inhibitor on the uptake rate of 100 μM l-tryptophan. Error bars indicate standard deviation. (C) Velocity of intracellular FLIPW-CTYT response versus external tryptophan concentrations used in (A) fitted with the Michaelis-Menten equation. Error bars indicate standard deviation. (D–F) Perfusion of KB cells with 100 μM l-tryptophan (L-Trp), 100 μM l-histidine (L-His), and 200 μM dl-kynurenine (DL-K) (D), 200 μM 3-hydroxy-dl-kynurenine (DL-HK) (E), and 1,000 μM 3-hydroxy-anthranilic acid (HAA) (F) in Tyrode's buffer. Cell images in panels (A) and (D–F) are pseudocolored to demonstrate the ratio change.

KB cells originate from a human oral squamous cell carcinoma and express only LAT1 for uptake of neutral amino acids as shown by reverse-transcriptase (RT)-PCR [37] and RNA interference (RNAi) [38]. When KB cells expressing FLIPW-CTYT were perfused with tryptophan, the FRET ratio increased (Figure 6A). The FRET ratio did not change upon subsequent perfusion with buffer, but it decreased upon perfusion with histidine, confirming amino acid exchange. LAT1-mediated uptake and exchange of tryptophan was similar to that observed for the LAT1-like exchanger in COS-7 cells (Figure 6A) and was almost completely inhibited by BCH (Figure 6B.). The affinity of KB cells for combined tryptophan uptake and metabolism under perfusion conditions is 5.1 ± 1.0 μM (Figure 6C), which is 2-fold lower than that observed for the LAT1-like exchanger of COS-7 cells. In addition, intracellular tryptophan was exported upon perfusion with kynurenine (Figure 6D), 3-hydroxy-kynurenine (Figure 6E), but not 3-hydroxy-anthranilic acid (Figure 6F), indicating that human LAT1 mediates the exchange of tryptophan for its kynurenine degradation products.

## Discussion

Mammalian cells cannot synthesize the amino acid tryptophan and rely on its import as tryptophan or as components of nutrients such as peptides across the plasma membrane for basic cell functioning. Tryptophan is necessary for protein synthesis, and it accounts for ∼1.3 % of the amino acids in human proteins. Tryptophan is also the precursor of other vital molecules like serotonin, melatonin, and NAD. Moreover, kynurenines produced from tryptophan appear to play a pivotal role in immunosuppression in inflammatory diseases and cancer [39,40].

The FLIPW nanosensors described in this study allow for noninvasive, real-time, spatio-temporal imaging of intracellular tryptophan levels and flux, offering advantages over conventional analytic methods. The E. coli transcriptional regulator TrpR was used as the recognition element for the construction of FRET sensors for tryptophan. As noted previously, the use of bacterial proteins for the construction of intracellular sensors reduces the problem of interference with endogenous metabolic and signal transduction pathways in eukaryotic cells [41]. Genetically encoded nanosensors further offer the advantage of subcellular sensor targeting through judicious choice of leader sequences as demonstrated with nuclear- and ER-targeted glucose nanosensors [42] and with cell-surface display of glutamate nanosensors [19]. Most FRET nanosensors are based on the ligand-binding–induced Venus fly trap–like conformational changes of bacterial PBPs [16–19], which consist of two well-structured lobes with the ligand-binding site located at the interface. TrpR is about three times smaller than the average PBP and is partially unfolded in the absence of tryptophan [25]. In the presence of tryptophan, the protein adopts the condensed conformation observed in crystal structures [22], and the concomitant conformational changes allow for the detection of tryptophan binding by FRET.

FRET has been a successful reporter signal for small molecule sensors [43,44]. According to the Förster theory, the efficiency of the energy transfer depends on the distance between the fluorophores and their dipole orientation [45]. These small molecule nanosensors can be engineered by modification of linker sequences between reporter and sensing domains and/or insertion of fluorophores in surface loops of the sensing domain, resulting in increased and/or reversed signal outputs of FRET nanosensors [29]. Because TrpR dimerizes to form its ligand-binding sites, we applied a novel approach for engineering of the FRET signal. Insertion of a second TrpR coding sequence in the principal FLIPW-CTY sensor changed the FRET response depending on the position of the insertion site. While insertion before eCFP and between eCFP and Venus almost eliminated the FRET response, a TrpR copy after Venus reversed the FRET response and increased the ratio change. Comparison of structural models of the FLIPW-CTY and FLIPW-CTYT sensors predicted that the fluorophores would be closer together in FLIPW-CTY. Since FRET efficiency is inversely correlated with the distance between the fluorophores as described in the Förster equation [45], the experimentally determined FRET ratio and the models are consistent.

FLIPW-CTYT was used for monitoring tryptophan uptake in cell cultures grown in 96-well microtiter plates, making the sensor suitable for high-throughput assays in which the effect of drugs or small interfering RNAs (siRNAs) is tested systematically [46]. The effective *K*_M_ for combined tryptophan uptake and metabolism in COS-7 cells in microplate assays and during perfusion was similar with values in the low micromolar range. We found that a LAT1-like transporter is responsible for the observed tryptophan exchange in COS-7 cells. Its transport and exchange characteristics are very similar to that of human LAT1 as expressed in KB cells. The reported affinity of human LAT1 for tryptophan uptake is 21 μM, about 4-fold lower than determined in this study [35]. However, the values are difficult to compare, because the former uptake experiments were performed under static conditions with LAT1 expressed in oocytes and the obtained affinities relate to the sum of intracellular pools of free, incorporated, and degraded tryptophan in the large oocytes. On the other hand, the affinities obtained using FLIPW-CTYT have been determined for the pool of free tryptophan in the targeted subcellular compartment, i.e., cytosol, under perfusion conditions with histidine as a defined counterexchange substrate. Possibly, the physiological conditions of amino acid exchange in the vascular system are mimicked more accurately by perfusion.

The transporters LAT1 and LAT2 are heteromeric obligatory counterexchangers of large, neutral amino acids with a 1:1 exchange stoichiometry (SLC3 and SLC7) [6,36]. As exchangers, they do not change the net intracellular amino acid concentration, but rather modify their relative concentrations. Perfusion of FLIPW-CTYT–expressing COS-7 and KB cells with tryptophan and histidine yielded high-resolution data of the real-time dynamics of free cytosolic tryptophan resulting from system L exchange activity. Importantly, we found that LAT transporters can exchange kynurenines and tryptophan. Since the individual intracellular and extracellular substrate selectivity of the LAT transporters are similar [36], kynurenine-tryptophan exchange may be bidirectional.

**Figure 7:**
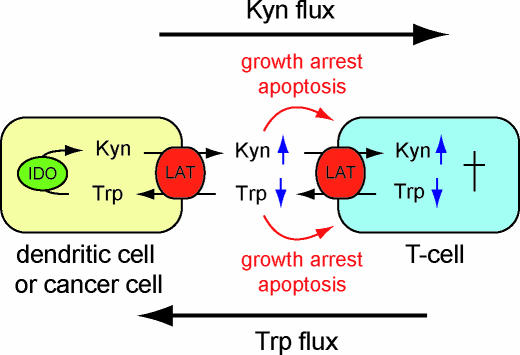
Double Trouble for T Cells Proposed model for the contribution of LAT-mediated tryptophan-kynurenine exchange to inflammation and immune escape. IDO- and LAT-expressing cell types such as dendritic cells or cancer cells replace tryptophan in the local environment with kynurenines. On the one hand, T cells, expressing LAT transporters for the transport of tryptophan [50], are drained of tryptophan; on the other hand, kynurenine levels increase. Both result in T cell growth arrest and apoptosis [39,40,51].

Tryptophan-kynurenine exchange may be part of an endogenous immunosuppressive mechanism during autoimmunity and may support the immune escape of proliferative cell types—like cancer cells—by enhancing the depletion of the local tryptophan pool and increasing the serum kynurenine concentrations (Figure 7). Kynurenines and hydroxy-kynurenine are natural amino acids that are produced from tryptophan through IDO, whose enzymatic activity is necessary for immune escape [13]. Macrophages and dendritic cells express IDO for endogenous suppression of the immune system [40,47]. Several human cancer cell lines, including KB cells, up-regulate IDO activity in the presence of the proinflammatory cytokine interferon-γ [48]. In addition, IDO can be up-regulated by the mutation of the tumor suppressor *Bin1* [14], which is lost or attenuated in several cancer types [49]. Intracellularly produced kynurenines serve as substrates for the exchange for extracellular tryptophan by LAT transporters, which are expressed in many tissues and human primary tumors [10]. Effectively, tryptophan is sequestered from the local environment and kynurenines accumulate in the serum. The kynurenines contribute to the pool of amino acids that can be taken up in exchange for intracellular tryptophan by surrounding cells expressing LAT transporters, a process which results overall in a tryptophan flux toward the IDO-producing cells. Since resting human T cells express only transporters of system L for the transport of l-tryptophan [50], the tryptophan-kynurenine exchange mechanism helps to deplete the intracellular tryptophan as well. Both the accumulation of kynurenines and depletion of tryptophan arrest T cell growth and induce apoptosis [39,40,51]. Thus, tryptophan-kynurenine exchange results in double trouble for T cells (Figure 7). At the same time, IDO-overproducing cells are protected from the apoptotic effect of kynurenines by the strict counterexchange of tryptophan and its stoichiometric degradation products. Cells, e.g., human macrophages, expressing transport systems with higher affinities for tryptophan than LAT1 will be able to continue to proliferate during ongoing tryptophan sequestration by the combined activity of LAT1 and IDO [50]. FLIPW sensors can now be used to test whether T cells take up kynurenines using the same pathway leading to a further drain of the essential tryptophan. The sensors can also be used to identify novel drugs and regulatory factors in genomic RNAi screens or screens of chemical libraries.

FRET nanosensors are unique tools for studying intracellular, small molecule steady-state levels and fluxes in vivo and in real time. Ultimately, complete metabolic routes can be monitored by using nanosensors that selectively detect single intermediates. For this means, a set of FRET nanosensors has been constructed that use the ligand-induced conformational changes of PBPs [16–19]. As the FLIPW sensors demonstrate, other protein scaffolds that undergo conformational changes upon ligand binding can provide sensing domains for nanosensors with specificities not represented in the PBP family, such as tryptophan. E. coli tryptophan repressor TrpR is not part of a protein family with different substrate specificities, which could be used for the expansion of the current set of nanosensors. However, the wealth of bacterial transcriptional regulators, which change affinity for operator sequences upon binding of effectors, may provide potential sensing domains for novel FRET metabolite nanosensors.

The FLIPW-CTYT nanosensor has proven to be a robust system with multiple advantages over conventional methods for intracellular tryptophan detection. The new sensor thus provides a complementary tool for monitoring steady state levels, uptake, and counterexchange, and will be an important tool for analyzing the factors that control tryptophan flux in living cells. As the kynurenine/tryptophan exchange demonstrates, such factors might contribute to important cellular processes such as inflammation and immune escape.

## Materials and Methods

### Chemicals, strains, plasmids.

All chemicals were of analytical grade and purchased from Sigma-Aldrich (http://www.sigmaaldrich.com). E. coli strains DH5α, TOP10 F′, and BL21(DE3)gold (Stratagene; http://www.stratagene.com) were used for transformation of Gateway reactions, cloning, and protein production, respectively.

### Construction of FLIPW sensors.

The *E. coli trpR* gene [52] (EcoGene EG11029, TrpR: UniProt P0A881) was amplified from genomic DNA by PCR for cloning in plasmid pGWF1 through pDONR using forward primer (5′-GGGGACAAGTTTGTACAAAAAAGCAGGCTCGGCCCAACAATCACCCTATTCAGC-3′) and reverse primer (5′-GGGGACCACTTTGTACAAGAAAGCTGGGTT ATCGCTTTTCAGCAACACCTCTTC-3′) using the Gateway protocol provided by the manufacturer (Invitrogen; http://www.invitrogen.com). Plasmid pGWF1 is based on the pRSETb expression vector (Novagen; http://www.novagen.com) and contains genes for eCFP and Venus cloned under control of the bacteriophage T7 promoter. Between the gene sequences of eCFP and Venus, a chloramphenicol resistance gene and lethal *ccdB* gene are flanked by *attP* DNA sequences for insertion of DNA sequences using Gateway cloning technology. The *trpR* gene was sandwiched between the eCFP and Venus coding sequences resulting in plasmid pTK164. The protein sequence encoded on pTK164 was denoted FLIPW-CTY. By PCR, *trpR* copies flanked with *Bam*HI or *Hind*III restriction site sequences were produced. Twin cassette sensor variants were constructed by insertion of *trp*R copies into pTK164 using unique *Bam*HI and *Hind*III restriction sites respectively before the eCFP coding sequence (resulting in pTK203) and after the Venus encoding sequence (resulting in pTK204), resulting in sensor variants encoding the repressor dimer in a single gene. A construct in which two *trp*R copies were connected with a Gly_7_ linker was denoted pTK205. The gene products of pTK203, pTK204, and pTK205 were denoted FLIPW-TCTY, FLIPW-CTYT, and FLIPW-CTTY, respectively. FLIPW constructs were harbored in E. coli BL21(DE3)gold, and sensor proteins were produced and purified as described previously [17].

### In vitro characterization of FLIPW sensors.

Purified sensor was added to a dilution series of ligand in 20 mM MOPS, pH 7.0, in the range of 10^−2^ to 10^−6^ M and analyzed in a monochromator microplate reader (Safire, Tecan; http://www.tecan.com; eCFP excitation 433/12 nm, eCFP emission 485/12 nm, and Venus emission 528/12 nm). eCFP shows two emission peaks at 476 nm and 501 nm [53]. The eCFP emission used for the ratio calculation was determined at 485 nm. Protein was diluted to give Venus readouts of 20,000–30,000 at a manual gain of between 70–75. By using the change in FRET ratio upon binding of ligand, affinity constants (*K*_d_) were determined by fitting the titration curves to a single-site binding isotherm:

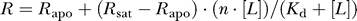
with [*L*], ligand concentration; *n*, number of equal binding sites; *R*, ratio; *R*_apo_, ratio in the absence of ligand; and *R*_sat_, ratio at saturation with ligand. Three independent protein preparations were analyzed and each protein preparation was analyzed in triplicate.

### 3D modeling of FLIP TrpR variants.

Structural models of FLIPW sensors were constructed using the crystal structures of Trp repressor in complex with L-Trp and Venus. Proteins were manually docked in the various topologies using MAGE (http://rd.plos.org/pbio.0050257).

### Tissue culture and transfection.

For cytosolic expression in COS-7 and KB cells, the gene encoding CTYT was amplified by PCR with primers encoding unique *Bam*HI and *Eco*RI restriction sites at the 5′ and 3′ end, respectively, and cloned into *Bam*HI/*Eco*RI digested pcDNA3.1(+) vector (Invitrogen), resulting in plasmid pTK222. COS-7 cells were grown in Dulbecco's modified Eagle's medium (high glucose; DMEM, Gibco; http://www.invitrogen.com) with 10% fetal calf serum and 50 units (U)/ml penicillin and 50 μg/ml streptomycin (Gibco). KB cells were grown in modified Eagle's medium alpha (MEM-α, Gibco) with 10% fetal calf serum and 50 U/ml penicillin and 50 μg/ml streptomycin (Gibco). Cells were cultured at 37 °C and 5% CO_2_. For imaging, cells were cultured in 8-well LabTekII German tissue culture glass slides (Nalge Nunc International; http://www.nalgenunc.com) and transiently transfected at 50%–70% confluence using Lipofectamine 2000 Reagent (Invitrogen) in Opti-MEM I reduced serum medium (Gibco). After transfection, cells were cultured for 16 h in Opti-MEM I followed by 3 h in growth medium prior to imaging. Transfection efficiency as determined by counting fluorescing cells was at least 30% for COS-7 and up to 5% for KB.

### Microplate assays.

Adherent COS-7 cells in 96-well microplates were washed once with 100 μl Tyrode's buffer (119 mM NaCl, 2.5 mM KCl, 2 mM CaCl_2_, 2 mM MgCl_2_, 25 mM HEPES, 30 mM glucose, pH 7.3–7.4). The initial FRET ratio was measured by recording the eCFP and Venus emissions at 485 nm and 528 nm, respectively, after excitation of eCFP at 433 nm in a Safire monochromator microplate reader (Tecan). Standard deviation of the initial ratios was less than 10%. After addition of 100 μl tryptophan in Tyrode's buffer the FRET ratio was recorded with 2-min intervals for up to 2 h. Uptake rates were determined from linear parts in the initial FRET change and fitted with the nonlinear regression program Origin 6.1 (OriginLab; http://www.originlab.com).

### Imaging.

Ratio imaging was performed on an inverted fluorescence microscope (DM IRE2, Leica; http://www.leica.com) with a Quant EM digital camera (Photometrics; http://www.photomet.com) and 20× oil immersion, 63× water immersion lenses (HC PL APO 20×/0.7 or HCX PL APO, Leica). Dual emission intensity ratios were simultaneously recorded using a DualView with an OI-5-EM filter set (eCFP 480/30; eYFP 535/40; Optical Insights; http://www.optical-insights.com) and Slidebook 4.2 (Intelligent Imaging Innovations; http://www.intelligent-imaging.com/). A Lambda DG4 (Sutter Instruments; http://www.sutter.com) provided excitation light. Images were acquired within the linear detection range of the camera and exposure times varied between 50 and 200 ms, depending on the expression level, with software binning between 2 and 3. Fluorescence intensities for eCFP and Venus were typically in the range of 6,000–8,000 and 12,000–16,000, respectively. Typical background values were around 1,000. Cells were perfused with Tyrode's buffer supplemented with ligands at flow rates of 1.0 ml/min in a vacuum chamber (Vacu-Cell VC-MPC-TW, C&L Instruments; http://www.fluorescence.com/) with a total volume of 0.1 ml. Buffers were exchanged using an eight-way automated ValveBank (AutoMate; http://www.autom8.com/). Inhibitors BCH and α-(methylamino)isobutyric acid (MeIAB) were used at 5 mM concentrations. For determination of the substrate specificity of tryptophan exchange, all 20 amino acids were tested at 100 μM concentrations. Analyses were repeated at least three times with similar results.

## Supporting Information

Figure S1 Ligand Specificity of FLIPW-CTY Normalized FRET ratio change of FLIPW-CTY in presence of l-tryptophan (red squares), d-tryptophan (cyan circles), 5-hydroxy-l-tryptophan (yellow squares), and 5-methyl-l-tryptophan (green triangles). Ratio is defined as fluorescence intensity quotient of emission at 528 nm/485 nm.
(39 KB DOC)

Figure S2 Relative Position of the Components of the FLIPW-CTYT Sensor The TrpR dimer (green, magenta) and Venus (yellow) are modeled to be sterically compatible, with the termini approaching within 1 Å.
(147 KB DOC)

Figure S3 Structural Models of FLIPW-TCTY and FLIPW-CTYT Tryptophan Sensors TrpR molecules are shown in green and magenta , eCFP is shown in blue (based on the Venus structure), and Venus is shown in yellow. (A) FLIPW-TCTY monomer, (B) FLIPW-CTYT monomer.
(496 KB DOC)

Table S1 Signal Change and l-Tryptophan Affinities of FLIPW Sensors (21 KB DOC)

Table S2 Affinities of FLIPW-CTY and FLIPW-CTYT for Tryptophan Substrates (mM) (20 KB DOC)

### Accession Numbers

The Protein Data Bank (http://www.pdb.org) accession numbers for structures discussed in this text are: 1TRO (Trp repressor with operator), 1MYW (Venus), and 1WRP (Trp repressor in complex with L-Trp).

## Abbreviations

BCH: 2-aminobicyclo-(2,2,1)-heptane-2-carboxylic acid
eCFP: enhanced cyan fluorescent protein
FLIPW: fluorescent indicator protein for detection of tryptophan
FRET: fluorescence resonance energy transfer
HK: 3-hydroxy-kynurenine
IDO: indoleamine 2,3-dioxygenase
K: kynurenine
FK: formylkynurenine
LAT: L-type amino acid transporter
PBP: periplasmic binding protein
TrpR: *Escherichia coli* tryptophan repressor
Venus: a yellow fluorescent protein variant
