# Supplementary material for: Nanosensor Detection of an Immunoregulatory Tryptophan Influx/Kynurenine Efflux Cycle
Source: PLoS Biol. 2007 Sep 25;5(10):e257. doi: 10.1371/journal.pbio.0050257 (PMC1988858; doi:10.1371/journal.pbio.0050257)
Supplement: Supplementary file 1 [file pbio.0050257.sg001.doc]

**Figure S1. Ligand specificity of FLIPW-CTY**

Normalized FRET ratio change of FLIPW-CTY in presence of l-tryptophan (red squares), d-tryptophan (cyan circles), 5-hydroxy-l-tryptophan (yellow squares) and 5-methyl-l-tryptophan (green triangles). Ratio is defined as fluorescence intensity quotient of emission at 528 nm/485 nm.
