# Supplementary material for: Nanosensor Detection of an Immunoregulatory Tryptophan Influx/Kynurenine Efflux Cycle
Source: PLoS Biol. 2007 Sep 25;5(10):e257. doi: 10.1371/journal.pbio.0050257 (PMC1988858; doi:10.1371/journal.pbio.0050257)
Supplement: Supplementary file 2 [file pbio.0050257.sg002.doc]

**Figure S2.** **Relative position of the components of the FLIPW-CTYT sensor**

The TrpR dimer (green, magenta, PDB: 1WRP) and Venus (yellow, PDB: 1MYW) are modeled to be sterically compatible, with the termini approaching within 1 Å.
