# Supplementary material for: Nanosensor Detection of an Immunoregulatory Tryptophan Influx/Kynurenine Efflux Cycle
Source: PLoS Biol. 2007 Sep 25;5(10):e257. doi: 10.1371/journal.pbio.0050257 (PMC1988858; doi:10.1371/journal.pbio.0050257)
Supplement: Supplementary file 3 [file pbio.0050257.sg003.doc]

**Figure S3. Structural models of FLIPW-TCTY and FLIPW-CTYT tryptophan sensors**

TrpR molecules are shown in green, magenta (PDB: 1WRP), eCFPis shown in blue (based on PDB: 1MYW) and Venus in yellow (PDB: 1MYW). (A) FLIPW-TCTY monomer, (B) FLIPW-CTYT monomer.
