# Supplementary material for: Nanosensor Detection of an Immunoregulatory Tryptophan Influx/Kynurenine Efflux Cycle
Source: PLoS Biol. 2007 Sep 25;5(10):e257. doi: 10.1371/journal.pbio.0050257 (PMC1988858; doi:10.1371/journal.pbio.0050257)
Supplement: Supplementary file 4 [file pbio.0050257.st001.doc]

**Table S1.** **Signal change and l-tryptophan affinities of FLIPW sensors**

| Sensor | **Apo ratio^*^** | **Δratio** | **Δratio (%)** | ***K*_d_ (μM)** |
| --- | --- | --- | --- | --- |
| FLIPW-CTY | 4.10 | -0.41 | -10 | 220 ± 20 |
| FLIPW-TCTY | 1.57 | -0.03 | -2 | 20 |
| FLIPW-CTYT | 2.17 | 0.75 | 35 | 100 ± 14 |
| FLIPW-CTTY | 2.85 | n.d. ^†^ | n.d. ^†^ | n.d. ^†^ |

^*^ ratio defined as fluorescence intensity quotient of emission at 528 nm/485 nm

^†^ n.d. not determined
