# Supplementary material for: Nanosensor Detection of an Immunoregulatory Tryptophan Influx/Kynurenine Efflux Cycle
Source: PLoS Biol. 2007 Sep 25;5(10):e257. doi: 10.1371/journal.pbio.0050257 (PMC1988858; doi:10.1371/journal.pbio.0050257)
Supplement: Supplementary file 5 [file pbio.0050257.st002.doc]

Table S2. Affinities of FLIPW-CTY and FLIPW-CTYT for tryptophan substrates (mM)

| Substrate | FLIPW-CTY | FLIPW-CTYT |
| --- | --- | --- |
| l-tryptophan | 0.22 ± 0.02 | 0.100 ± 0.014 |
| d-tryptophan | 3.1 ± 0.3 | 1.28 ± 0.10 |
| l-5-methyl-tryptophan | 0.06 ± 0.01 | 0.025 ± 0.002 |
| l-5-hydroxy-tryptophan | 6.0 ± 0.8 | n.d.^*^ |

^*^ n.d. not determinable, data could not be fitted with a formula for single site binding
